# Association of Ozone with 5-Fluorouracil and Cisplatin in Regulation of Human Colon Cancer Cell Viability: In Vitro Anti-Inflammatory Properties of Ozone in Colon Cancer Cells Exposed to Lipopolysaccharides

**DOI:** 10.1155/2017/7414083

**Published:** 2017-07-04

**Authors:** Vincenzo Simonetti, Vincenzo Quagliariello, Pierangela Giustetto, Marianno Franzini, Rosario Vincenzo Iaffaioli

**Affiliations:** ^1^“Kaos” ONLUS Foundation, Turin, Italy; ^2^Oxygen-Ozone Therapy Scientific Society (SIOOT), Gorle, Italy; ^3^Oncology Department, Istituto Nazionale Tumori, IRCCS, Fondazione G. Pascale, Naples, Italy; ^4^Freedom Waves Srl, Via Sicilia 31, 42122 Reggio Emilia, Italy; ^5^University of Pavia, Pavia, Italy

## Abstract

**Introduction:**

Ozone therapy is an effective medical treatment for different diseases like mucositis, psoriasis, acute pain, neurovascular diseases, and cancer. The aim of this study is based on the association of different ozone concentration with 5-fluorouracil and cisplatin in human colon cancer cell (HT29 cell line) in order to investigate possible anticancer synergistic effects.

**Methods:**

HT29 cells were incubated with ozone at different concentration ranging from 10 up to 50 *μ*g/ml at different incubation time alone or in combination with cisplatin and 5-fluorouracil. Cell viability was performed by using a modified MTT method. Anti-inflammatory studies were conducted incubating HT29 with or without 20, 30, or 50 *μ*g/ml of ozone before exposure to lipopolysaccharides.

**Results:**

Ozone alone has a time and concentration dependent cytotoxicity against HT29 cells (IC50 at 24 h: 30 *μ*g/ml). Association of ozone with drugs increases cytotoxicity by 15–20%. Preincubation of ozone at 50 *μ*g/ml decreases IL-8, IL-6, and IL-1*β* production by 50, 56, and 70%, respectively, compared to untreated cells.

**Conclusion:**

These results indicated that ozone could be useful in colon cancer management in combination with 5-fluorouracil and cisplatin with significant inhibition of cytokines having a central role in colon cancer cell survival and chemoresistance.

## 1. Introduction

In the last years, the use of ozone as an alternative tool for management of several clinical disorders was sharply increased [1]. Ozone therapy is a nonconventional form of medicine that has been used successfully in the treatment of ischemic disorders; as example, chronic middle ear deafness and tuberculosis are two diseases well treated by using ozone [2, 3]. In oncology, the use of ozone was well known in order to stimulate tissue oxygenation [4]; in fact, despite being administered over a very short period, ozone therapy improved oxygenation in hypoxic tumors in same clinical studies [4]. Specifically, ozone has several putative mechanisms of action involving, for example, nuclear factor-erythroid 2-related factor 2 (Nrf2), a nuclear transcriptional factor with interesting anticancer and protective actions against neurodegenerative diseases like Alzheimer's and Parkinson's diseases [5]. In fact, the activation of Nrf2 by ozone could increase the activity of several tumor suppressor proteins like SOD, catalase (CAT), GSH, GPx, GSH-S-transferase (GSTr), NADPH quinine-oxidoreductase 1 (NQO1), and heat shock protein 70 (HSP70). These effects are substantially comparable to those observed with the use of sulforaphane, a common natural molecule found in broccoli and other green vegetables, or curcumin with interesting anticancer activities in particular related to breast cancer chemoprevention [6, 7]. One of the most important factors involved in cancer growth, survival, and resistance to many chemotherapeutics is the anoxic microenvironment; from a clinical point of view, tumor hypoxia is an independent prognostic factor for advanced cancer progression [8]; in fact patients with hypoxic tumors have significantly lower overall survival or disease-free survival [9]. One of the most interesting properties of ozone therapy is obviously the impact on the tissue oxygenation; in fact, as recently well demonstrated in a pilot study, ozone therapy could increase oxygenation in several hypoxic tumor tissues and it could be useful as possible adjuvant in chemoradiotherapy regimens [4]. Despite the recent clinical studies related to ozone therapy, more biological and biochemical studies are required in order to understand the limitations and the possible adverse effects related to its use in human. However, in view of potential role of ozone in cancer treatment, we were interested to demonstrate if ozone could determine cell death in human colon cancer cell line and its possible anti-inflammatory properties based on modulation of cytokines secretion by cancer cells, considering the central role of several small molecules like IL-8 and IL-6 in colon cancer cell survival and chemoresistance [10].

## 2. Materials and Methods

### 2.1. Cell Viability

The cytotoxicity of ozone, 5-FU, and cisplatin was evaluated on human colon cancer cells looking at their mitochondrial dehydrogenase activity by means of a modified MTT [3-(4,5-dimethylthiazol-2-yl)-2,5-diphenyltetrazolium bromide] method according to the manufacturer's instructions (Dojindo Molecular Technologies Inc., Rockville, MD).

HT-29 (ATCC® HTB-38™) human colon cancer cell line was grown in McCoy's 5a Medium Modified supplemented with 10% FBS and 1% Pen-Strep and seeded in 96-well plates at a density of 10000 cells per well at 37°C in a humidified 5% CO2 atmosphere.

After 24 h of appropriate growth, we tested the following solutions in full medium and added them to the cells after washing them with PBS: ozone (from 10 up to 50 *μ*g/mL); cisplatin (from 5 up to 1500 *μ*M) alone or in combination with ozone 10, 20, 30, and 50 *μ*g/mL; 5-FU (from 0.1 up to 100 *μ*M) alone or with ozone (produced with Multiossigen machinery, type Medical 99 IR) 10, 20, 30, and 50 *μ*g/mL. Specifically, ozone treatment to HT-29 cells was performed following the same procedure reported in literature [11] by a method that avoided cells from direct expose to ozone.

After treatments, cancer cells were then incubated for 24 h under standard conditions. At the end of the incubation period the cells were washed three times with PBS at pH 7.4 and incubated with 100 *μ*l of a MTT solution (0.5 mg/ml in cell culture medium) for 4 h at 37°C. The absorbance readings were acquired at a wavelength of 450 nm with the Tecan Infinite M200 plate reader using I-control software. The relative cell viability (%) was calculated by the formula [*A*] test/[*A*] control × 100, where “[*A*] test” is the absorbance of the test sample and “[*A*] control” is the absorbance of the control cells incubated solely within culture medium. After evaluating cell cytotoxicity, the total protein content was measured by using the Micro BCA protein assay kit (Pierce). Briefly, the cells were washed with ice-cold PBS and incubated for 15 min in 150 *μ*l cell lysis buffer (0.5% v/v Triton X-100 in PBS), to which 150 *μ*L of Micro BCA protein assay kit reagent (prepared following the instructions of the manufacturer) was added. The absorbance at 562 nm was finally measured on a plate reader. The cytotoxicity measurements were then normalized by the amount of total protein content in each well.

### 2.2. Anti-Inflammatory Tests

The expression of IL-6, IL-8, and IL-1*β* by human colorectal cells was evaluated with ELISA, as described in literature [12, 13]. Briefly, HT-29 cells (1.2 × 10^5^ cells/well) were seeded in 12-well plate in McCoy's 5a Medium Modified supplemented with 10% FBS and 1% Pen-Strep at 37°C in a humidified 5% CO2 atmosphere. After preincubation for 24 h and starvation in serum-free medium for 2.5 h, the cells were treated with or without 0.1 ml of a full cell culture medium added with 20, 30, and 50 *μ*g/mL of ozone for 5 h before exposure to LPS (40 ng/ml) for 12 h, in order to stimulate inflammation. After that, culture supernatants were collected, centrifuged to pellet any detached cells, and measured using an IL-1*β* ELISA Kit (Sigma Aldrich, Milan, Italy) or an IL-6 and IL-8 ELISA Kits (Sigma Aldrich, Milan, Italy). The ELISAs were performed according to the manufacturer's instructions. The sensitivity of this method was less than 10 (pg/ml), and the assay can accurately detect cytokines in the range of 1–32000 pg/ml.

### 2.3. Statistical Analysis

The difference between experimental groups was investigated by a one-way analysis of variance (ANOVA) and by a subsequent Turkey's multiple comparison test in Sigma Plot Software. For statistical analysis of all data, *p* < 0.05 was regarded as the lowest acceptable threshold for significance.

## 3. Results

### 3.1. Cell Viability

**Figure 1:**
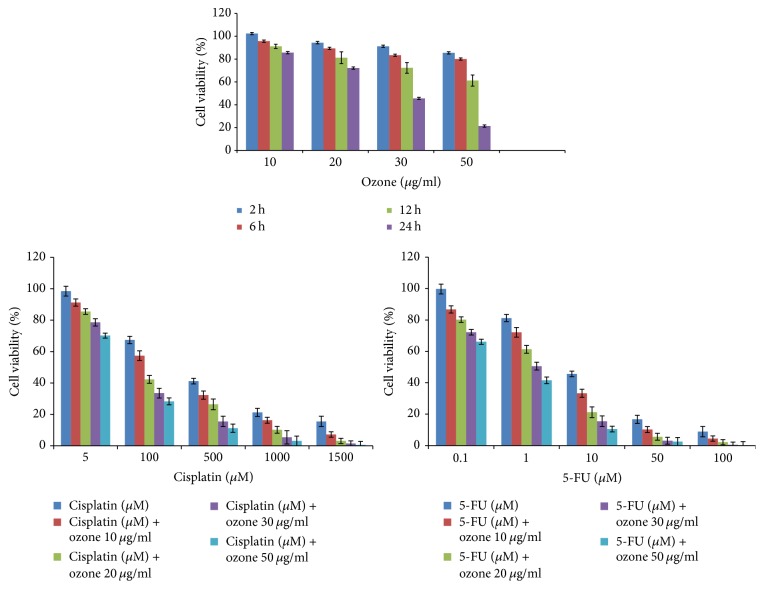
HT29 cell viability (±SEM) performed by modified MTT method as in function of the time (from 2 h up to 24 h) and ozone concentration (from 10 up to 50 *μ*g/ml) and only in function of the concentration of anticancer drugs (cisplatin tested from 5 up to 1500 *μ*M; 5-fluorouracil tested from 0.1 up to 100 *μ*M) alone and in combination with ozone after 24 h of incubation.

As clearly shown in Figure 1, ozone affected in a time and dependent manner the cell viability of colon cancer cells. Specifically, after 12 h of incubation, ozone reduced around 40% cell viability compared to control cells at 50 *μ*g/mL (*p* < 0.01). Instead after 24 h of incubation, ozone showed an IC50 value of around 30 *μ*g/ml. Regarding anticancer drugs tested alone or in combination with ozone after 24 h of incubation, we have seen, as reported in literature, that cisplatin and 5-FU alone have an IC50 value of around 200–250 *μ*M and 10 *μ*M, respectively. When coincubated with ozone both drugs have higher cytotoxicity against HT-29 but these effects are statistically significant only from 20 up to 50 *μ*g/ml of ozone (Figure 1). Specifically, as example, IC50 value of cisplatin associated with ozone at 20 and 50 *μ*g/ml was detected at around 100 and 70 *μ*M, respectively, and regarding 5-FU its IC50 value associated with ozone at 20 and 50 *μ*g/ml was observed at around 5 and 2 *μ*M, respectively, after 24 h of incubation.

### 3.2. Measurement of Cytokines Analysis

**Figure 2:**
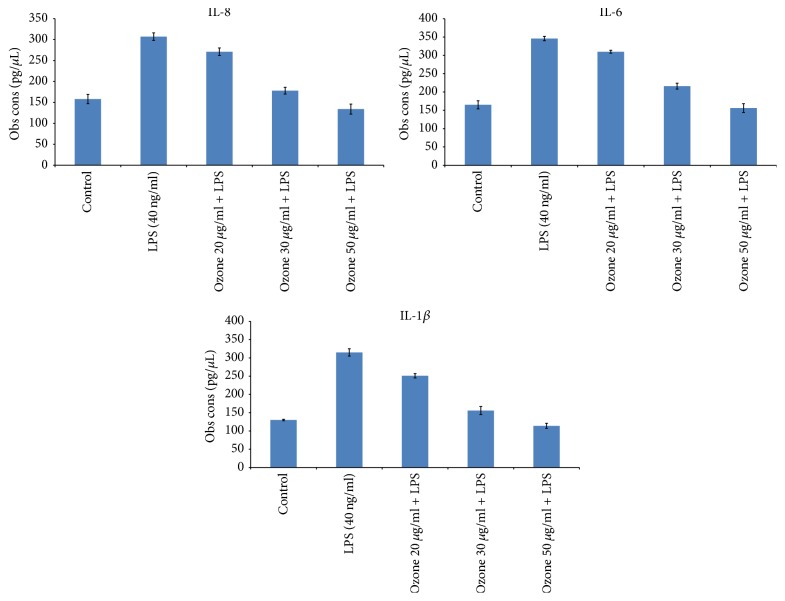
Anti-inflammatory properties of ozone based on affections of IL-8, IL-6, and IL-1*β* production on HT29 cell line (1.2 × 10^5^ cells/well). Cells were pretreated with or without ozone at 20, 30, and 50 *μ*g/ml for 5 h before exposure to LPS (40 ng/ml) for 12 h.

Considering the possible anti-inflammatory activity of ozone, we have investigated the possible effects on the IL-8, IL-6, and IL-1*β* production in HT-29 cells under proinflammatory conditions, incubating them with LPS at a dose of 40 ng/ml. First of all, incubation of HT-29 cells only with LPS determines a significant stimulation of all analyzed interleukins compared to untreated cells due to the stimulation of Toll Like Receptor type 4 (TLR4) expressed on the membrane of human colon cancer cells leading to an upregulation of interleukins expression mRNA and their secretion [14]. Pretreatment with ozone at all tested concentrations decreased significantly the level of all analyzed interleukins (Figure 2) and the effects are concentration dependent. Specifically, ozone pretreatment at 20 *μ*g/ml reduced the magnitude of the increase in IL-8, IL-6, and IL1-*β* cellular levels approximately by 16, 10, and 21%, respectively, compared to unpretreated, only LPS stimulated, cells (*p* < 0.05) (Figure 2). Moreover, ozone pretreatment at 50 *μ*g/mL reduced the magnitude of the increase in IL8, IL6, and IL1-*β* cellular levels approximately by 60, 56, and 68%, respectively, compared to unpretreated, only LPS stimulated, cells (*p* < 0.001) (Figure 2).

## 4. Discussion

The ozone therapy is an alternative therapeutic method of extreme interest in various fields of medicine. Although ozone is a powerful direct cellular oxidant, it appears to be a stimulator of the antioxidant defenses of the human organism as widely discussed in the literature (called ozone paradox effect) [15, 16]. Interestingly, it has been observed that several effects of ozone therapy are clinically similar to hyperbaric oxygen [16]. Local uses of ozone could improve radiation-induced side effects in patients [17, 18]. It is well demonstrated how ozone therapy could enhance the oxygenation of several healthy and pathological tissue like muscle [19–21]. Biological effects of ozone can be enhanced and applicable in topic manner by using ozone-enriched oils [22–24] specifically that of sesame or olive oil that are able to manage radiation proctitis as well as increase cutaneous wound healing processes.

From a mechanistic point of view, the biological effects of ozone are not related to a specific membrane or intracellular receptor; in fact it acts as inducer of oxidative stress firstly on antioxidants and membrane polyunsaturated fatty acids (PUFA) in a process called lipidic peroxidation [5]. This mechanism leads to a production of second messengers called hydrogen peroxide (H_2_O_2_) and alkenals (mainly 4-hydroxynonenal, 4-HNE) both able also to activate the nuclear factor- (erythroid-derived 2) like 2 (Nrf2); this activation determines antioxidant and anti-inflammatory effects based principally on the induction of superoxide dismutase, glutathione-peroxidase, heat shock proteins (HSP-70), and heme oxygenase-1 (HO-1). Regarding the anti-inflammatory effects of ozone, it could be related to Nrf2 that can lead to suppression of nuclear factor kappa B (NF*κ*B), the most important transcriptional factor involved in several inflammatory as well as cancer metabolisms. The anti-inflammatory activities of ozone arouse great interest for a possible modulation of colon cancer microenvironment providing interesting insights for further studies, also in combination with common anticancer drugs. This could be valuable especially considering the crucial role of interleukins in the progression, metastatic process, and drug resistance of human cancer cells. Specifically, it is interesting that interleukins like 6 and 8 act by forming a complex with region alpha of their receptors and a glycoprotein called gp130 with activation of the signal transducer and activator of transcription (STATs) able to stimulate cell division and survival of fibroblasts and tumor growth in breast, colon cancer, and glioma [25, 26]. Clinically, as recently demonstrated, IL-6 and IL-8 levels correlate with histologic grade in several cancers and specifically with neovascularization processes [27]. Another clinical crucial aspect is based on the observation that cancer patients affected by Metabolic Syndrome (MS), having conventionally a poor prognosis and increased cancer mortality with a greater relative risk of neoplastic recurrence compared to patients without MS, have higher serum levels of interleukins and cytokines as well as hormones involved in chemoresistance processes [28] so the possible modulation of the tissue concentration of these cytokines may play a central role in the therapeutic management of the disease. The results of the this study demonstrate the possible interesting effects of ozone associated with common anticancer drugs cisplatin and 5-FU. On the basis of these findings, we can argue that the combined use of the ozone with both common anticancer agents increases cell cytotoxicity of human colon cancer cells. However, one limitation of our work is based on the absence of cell death studies (necrosis or apoptosis) and a critical comparison with the biological effects of ozone in noncancer cells (as fibroblasts or endothelial cells). On the basis of this consideration we are extremely cautious to assess that ozone could be totally safe in the treatment of certain chronic diseases because more biological studies are required. Anyway, the overall picture offered by these experiments shows how ozone could have a great potential as alternative treatment in association with common anticancer drugs for colon cancer treatment with interesting abilities based on the inhibition of interleukins having a key role in cancer cell survival, progression, and resistance to chemotherapy opening a possible window in the management of colon cancer.
