# Corrigendum to “Association of Ozone with 5-Fluorouracil and Cisplatin in Regulation of Human Colon Cancer Cell Viability: In Vitro Anti-Inflammatory Properties of Ozone in Colon Cancer Cells Exposed to Lipopolysaccharides”

**DOI:** 10.1155/2018/3297906

**Published:** 2018-04-30

**Authors:** Vincenzo Simonetti, Vincenzo Quagliariello, Pierangela Giustetto, Marianno Franzini, Rosario Vincenzo Iaffaioli

**Affiliations:** ^1^“Kaos” ONLUS Foundation, Turin, Italy; ^2^Oxygen-Ozone Therapy Scientific Society (SIOOT), Gorle, Italy; ^3^Oncology Department, Istituto Nazionale Tumori, IRCCS, Fondazione G. Pascale, Naples, Italy; ^4^Freedom Waves Srl, Via Sicilia 31, 42122 Reggio Emilia, Italy; ^5^University of Pavia, Pavia, Italy

In the article titled “Association of Ozone with 5-Fluorouracil and Cisplatin in Regulation of Human Colon Cancer Cell Viability: In Vitro Anti-Inflammatory Properties of Ozone in Colon Cancer Cells Exposed to Lipopolysaccharides” [1], there was a missing affiliation and an error in the third affiliation, the authors' full first names were not included, the Authors' Contributions were mistakenly included as Acknowledgements, and the correct Acknowledgements were not included. There were also errors in the Materials and Methods and Conflicts of Interest sections.

(i) The revised authors' names and affiliations are shown above and have been corrected in place.

(ii) In Materials and Methods, the wording “alone or in combination with ozone 10, 20, 30, and 50 *μ*g/mL” should be corrected to “alone or with ozone (produced with Multiossigen machinery, type Medical 99 IR) 10, 20 30, and 50 *μ*g/mL.”

(iii) An Authors' Contributions section should be added as follows.

## Authors' Contributions

Dr. Pierangela Giustetto provided useful suggestions and statistical studies related to the ozone treatment. Professor Vincenzo Simonetti provided suggestions related to experience with ozone treatments. Professor Rosario Vincenzo Iaffaioli managed the pharmacological data related to colon cancer treatments. Dr. Vincenzo Quagliariello conducted cellular experiments related to the biological effects of ozone in colon cancer cells (cell viability and anti-inflammatory tests) with subsequent statistical analysis. Professor Marianno Franzini coordinated the overall work plan and the choice of type of experiments to be performed in experimental work.

(iv) The Acknowledgements section should be updated as follows:

  The authors thank Oxygen-Ozone therapy Scientific Society (SIOOT) for assistance in terms of research and materials throughout the scientific study.

(v) The Conflicts of Interest sections should be updated as follows:

  The authors declare that Dr. Pierangela Giustetto is an employee at Freedom Waves Srl but has no interest in ozone therapy for the treatment of any pathology. The authors also declare that Dr. Vincenzo Simonetti is the President of the “Kaos” Onlus Association and a board member of SIOOT, and Dr. Marianno Franzini is the President of SIOOT.

(vi) All the aforementioned corrections have been applied in place.
